# Carbohydrate Deacetylase Unique to Gut Microbe Bacteroides Reveals Atypical Structure

**DOI:** 10.1021/acs.biochem.4c00519

**Published:** 2024-12-12

**Authors:** Lilith A. Schwartz, Jordan O. Norman, Sharika Hasan, Olive E. Adamek, Elisa Dzuong, Jasmine C. Lowenstein, Olivia G. Yost, Banumathi Sankaran, Krystle J. McLaughlin

**Affiliations:** † Department of Chemistry, Vassar College, 124 Raymond Ave, Poughkeepsie, New York 12604, United States; ‡ Biochemistry Program, Vassar College, 124 Raymond Ave, Poughkeepsie, New York 12604, United States; § Molecular Biophysics and Integrated Bioimaging, Berkeley Center for Structural Biology, Lawrence Berkeley National Laboratory, Berkeley, California 94720, United States

## Abstract

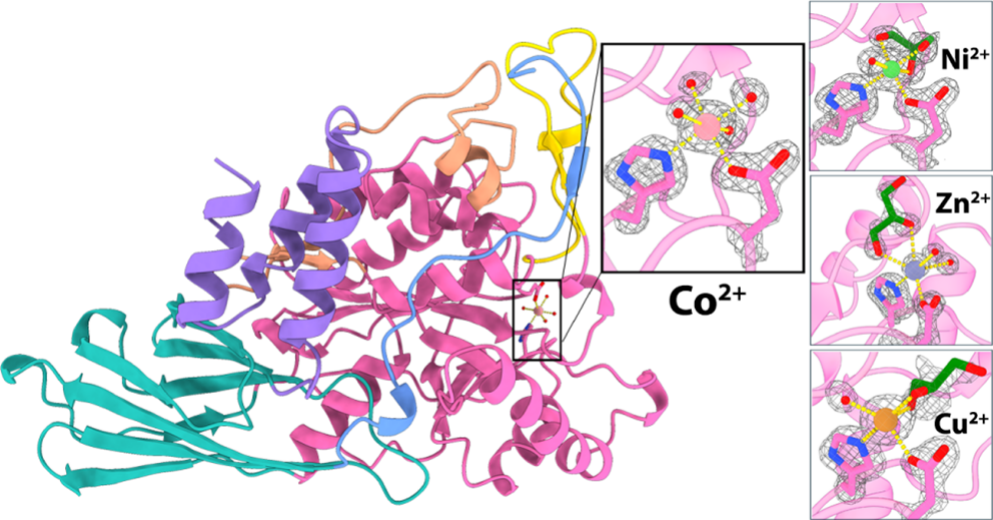

*Bacteroides* are often the most abundant, commensal species in the gut microbiome of industrialized human populations. One of the most commonly detected species is *Bacteroides ovatus*. It has been linked to benefits like the suppression of intestinal inflammation but is also correlated with some autoimmune disorders, for example irritable bowel disorder (IBD). Bacterial cell surface carbohydrates, like capsular polysaccharides (CPS), may play a role in modulating these varied host interactions. Recent studies have begun to explore the diversity of CPS loci in *Bacteroides*; however, there is still much unknown. Here, we present structural and functional characterization of a putative polysaccharide deacetylase from *Bacteroides ovatus* (*Bo*PDA) encoded in a CPS biosynthetic locus. We solved four high resolution crystal structures (1.36–1.56 Å) of the enzyme bound to divalent cations Co^2+^, Ni^2+^, Cu^2+^, or Zn^2+^ and performed carbohydrate binding and deacetylase activity assays. Structural analysis of *Bo*PDA revealed an atypical domain architecture that is unique to this enzyme, with a carbohydrate esterase 4 (CE4) superfamily catalytic domain inserted into a carbohydrate binding module (CBM). Additionally, *Bo*PDA lacks the canonical CE4 His-His-Asp metal binding motif and our structures show it utilizes a noncanonical His-Asp dyad to bind metal ions. *Bo*PDA is the first protein involved in CPS biosynthesis from *B. ovatus* to be characterized, furthering our understanding of significant biosynthetic processes in this medically relevant gut microbe.

## Introduction

Bacteroides are a major constituent of the human gut microbiome.^1,2^ Dietary patterns and geography impact the prevalence of Bacteroides observed in the gut, and in industrialized populations Bacteroides can make up 25–40% of the gut bacteriome.^1−5^ Generally *Bacteroides* spp. are mutualistic, playing crucial beneficial roles like complex carbohydrate catabolism, supplying nutrients to other microbial residents of the gut, and shielding the host from pathogens.^2^ However, Bacteroides can also be associated with disease states, such as autoimmune disorders like Crohn’s disease and colorectal cancers resulting from dysbiosis of the gut.^1,2^

Two of the most well characterized *Bacteroides* spp. are *B. fragilis* and *B. thetaiotaomicron*.^1,2^*B. fragilis* is a highly clinically relevant species, and *B. thetaiotaomicron* has emerged as a model organism for the gut microbiota.^1,2^ Both have been found to confer host benefits, for example *B. thetaiotaomicron* has approximately 90 polysaccharide utilization loci (PUL) allowing it to degrade a wide variety of carbohydrates from the human diet.^6^ Polysaccharide A (PSA) from *B. fragilis* is a zwitterionic cell surface capsular polysaccharide (CPS) that has known immunomodulatory effects, playing a key role in the development of the mature human immune system.^7,8^ Conversely, *B. fragilis* is the most common *Bacteroides* spp. isolate from intra-abdominal abscesses, and some *B. fragilis* strains encode the toxin fragilysin.^9−11^*B. thetaiotaomicron* has also been associated with various disease states including autoimmune inflammatory cardiomyopathy.^2,12^

*B. ovatus* has also emerged as a species of interest as *B. ovatus*, *B. fragilis* and *B. thetaiotaomicron* are three of the most commonly detected *Bacteroides* spp.^2^*B. ovatus* has also been implicated in playing dual mutualistic and pathogenic roles, though it is not as well characterized as *B. fragilis* and *B. thetaiotaomicron*.^1,2^ Recent research demonstrated that short chain fatty acids (SCFAs) produced from carbohydrate fermentation in *B. ovatus* increased production of several neuro-active compounds such as GABA.^13^*B. ovatus* has been linked to a variety of autoimmune disorders such as inflammatory bowel disorder (IBD) and type I diabetes.^2,14,15^ Additionally, *B. ovatus* has shown promise as a bacteriotherapy treatment for various gastrointestinal diseases, serving as an alternative to fecal matter transplant (FMT), and as a candidate for an antitumor vaccine.^16−18^

Many of these host interactions are mediated in part by carbohydrates on the bacterial outer membrane, such as capsular polysaccharides (CPS).^19−22^ These complex polysaccharides improve bacterial fitness in several ways, including helping protect the expressing bacterium from fluctuating environmental conditions, and enhancing resistance to some antimicrobials.^19^ As a benefit to the host, CPS have been shown to prime the immune system for protection against invading pathogens, however their expression may contribute to disease states like autoimmune diseases.^2,22,23^ PSA from *B. fragilis* is the most well studied Bacteroides CPS, and in addition to its immunomodulatory effects, its carbohydrate structure and biosynthetic loci have been identified.^8,24−26^ Most Bacteroides, including *B. fragilis*, have been found to harbor multiple CPS loci but few enzymes from these loci have been characterized.^27^

Given the ubiquity of *B. ovatus* in the human gut and its developing therapeutic potential, it is important to gain a better understanding of its basic metabolic pathways, such as capsular polysaccharide biosynthesis. Here we present structural and functional characterization of a putative polysaccharide deacetylase from *Bacteroides ovatus* (*Bo*PDA) located in a capsular polysaccharide biosynthetic locus. *Bo*PDA represents the first protein involved in CPS biosynthesis from *B. ovatus* to be investigated. We solved four high resolution (1.36–1.56 Å) crystal structures of *Bo*PDA bound to different metal cations (Co^2+^, Ni^2+^, Zn^2+^ and Cu^2+^). The structures reveal an unusual nonmodular domain architecture for this enzyme that is distinct to the Bacteroides genus. Structural analyses of the active site along with carbohydrate binding and deacetylase activity assays provide insight into the preferred metal for enzymatic activity.

## Materials and Methods

### Expression and Purification of *Bo*PDA

*Bacteroides ovatus* polysaccharide deacetylase (*Bo*PDA; BACOVA_3992 from *Bacteroides ovatus* ATCC 8483 (taxonomy_id:411476) cloned into the pSpeedET vector expressing a His-tagged N-terminal truncated construct (residues 21–499) was obtained from the DNASU Plasmid Repository.^28−30^ BL21 (DE3) pLysS were transformed with the plasmid and cells were grown in Terrific Broth (TB) at 37 °C with 40 μg/mL of kanamycin, shaking at 200 rpm. When an OD_600_ of 0.8 was reached, the temperature was reduced to 17 °C and induced by adding 0.2 mM IPTG and 0.005% arabinose. Cells were harvested after 18 h via centrifugation and stored at −80 °C.

Purification of *Bo*PDA was conducted using nickel affinity chromatography and size exclusion chromatography (SEC). Cell pellets were thawed on ice then resuspended in lysis buffer (50 mM HEPES pH 8.0, 50 mM NaCl, 10 mM imidazole, 1 mM TCEP, 1.6 mg/mL lysozyme, 0.05 mg/mL DNase I). After addition of a Pierce protease-inhibitor tablet (Thermo Fisher Scientific), cells were sonicated then clarified via centrifugation at 15,000*g* for 0.5 h at 4 °C. Lysate was flowed over a HisTrap HP 5 mL column (Cytiva) equilibrated with Buffer B (50 mM HEPES pH 8.0, 50 mM NaCl, 10 mM imidazole, 1 mM TCEP). His-tagged protein was eluted using a gradient over 10 CV of Buffer E (50 mM HEPES pH 8.0, 50 mM NaCl, 300 mM imidazole and 1 mM TCEP). To remove the His-tag, peak fractions were dialyzed overnight into Buffer D (20 mM HEPES pH 8.0, 200 mM NaCl, 40 mM imidazole, 5% glycerol, 1 mM DTT) in the presence of tobacco etch virus (TEV) protease. Dialyzed protein was concentrated then sterile filtered at 0.22 μM before being injected onto a HiLoad 16/600 Superdex 200 pg SEC column (Cytiva) equilibrated with Buffer C (20 mM HEPES pH 8.0, 200 mM NaCl, 40 mM imidazole, 1 mM TCEP). To allow estimation of the protein’s molecular weight (*M*_r_) the SEC column was calibrated using the Gel Filtration Calibration HMW Kit (Cytiva), generating a calibration curve as previously described.^31,32^ Following SDS-PAGE analysis, pure SEC peak fractions were combined and the protein was concentrated to 23.6 mg/mL using a 30 kDa MWCO Amicon Ultra-15 Centrifugal Filter (Millipore). Purified *Bo*PDA was flash frozen in liquid nitrogen and stored at −80 °C.

### Crystallization, Structure Solution and Refinement

*Bo*PDA was crystallized using hanging drop vapor diffusion at 4 °C. Drops were 2 μL, formed from a 1:1 ratio of protein at 16.5 mg/mL to well solution (0.17 M NaOAc, 0.1 M Tris-HCl pH 8.5, 15% glycerol, and 25.5–27% PEG 4000). Crystals were soaked with either 1 mM ZnCl_2_, CoCl_2_, NiCl_2_ or CuCl_2_ then flash frozen in liquid nitrogen. Diffraction data sets were collected through the Collaborative Crystallography program in the Berkeley Center for Structural Biology at Advanced Light Source (ALS) beamline 8.2.2. All data sets were native, collected on single crystals at 100 K using wavelengths of 1.000 or 0.976 Å.

Diffraction data were processed and scaled with XDS^33^ and Pointless and Aimless in the CCP4 Suite.^34^ Molecular replacement with PDB entry 4dwe (Joint Center for Structural Genomics, unpublished work) as the starting model was run in Phaser^35^ to solve the structure. Iterative refinement and model building of each structure was completed in Phenix^36^ and Coot^37^ respectively. Data collection and final refinement statistics are provided in Table 1. All structural figures were rendered with ChimeraX.^38^

**Table 1:** Data Collection and Refinement Statistics^a^

	*Bo*PDA-Co^2+^	*Bo*PDA-Ni^2+^	*Bo*PDA-Zn^2+^	*Bo*PDA-Cu^2+^
PDB accession code	9D44	9D4I	9D60	9D5T
Data Collection				
diffraction source	ALS Beamline 822	ALS Beamline 822	ALS Beamline 822	ALS Beamline 822
wavelength (Å)	1.000	1.000	1.000	0.9765
resolution (Å)	47.71–1.42 (1.47–1.42)	44.36–1.48 (1.53–1.48)	47.69–1.36 (1.41–1.36)	47.71–1.56 (1.62–1.56)
space group	P 2_1_ 2_1_ 2_1_	P 2_1_ 2_1_ 2_1_	P 2_1_ 2_1_ 2_1_	P 2_1_ 2_1_ 2_1_
cell dimensions				
*a, b, c* (Å)	69.18, 73.16, 95.42	68.95, 73.04, 95.16	69.29, 73.11, 95.39	68.76, 73.18, 95.41
α, β, γ (deg)	90, 90, 90,	90, 90, 90,	90, 90, 90,	90, 90, 90,
total reflections	841069 (84015)	687244 (68163)	1082788 (102922)	658639 (67904)
unique reflections	91845 (9050)	80482 (7915)	100382 (9259)	68700 (6770)
multiplicity	9.2 (9.3)	8.5 (8.6)	10.8 (11.1)	9.6 (10.0)
completeness (%)	99.93 (100.0)	99.70 (99.17)	96.06 (90.11)	99.28 (99.50)
(*I*/σ(*I*))	16.37 (2.46)	19.95 (2.47)	35.50 (11.54)	15.25 (2.24)
Wilson B-factor	12.8	15.4	10.75	16.2
*R*_merge_	0.1118 (1.342)	0.07219 (0.9738)	0.0410 (0.186)	0.1172 (1.119)
*R*_pim_	0.03895 (0.4626)	0.02626 (0.3504)	0.0131 (0.058)	0.03968 (0.3674)
CC_1/2_	0.998 (0.807)	0.999 (0.778)	1 (0.987)	0.998 (0.817)
Refinement				
*R*_work_	0.1599 (0.2221)	0.1663 (0.2818)	0.1650 (0.1833)	0.1746 (0.2165)
*R*_free_	0.2007 (0.2857)	0.1932 (0.3022)	0.1768 (0.2113)	0.2005 (0.2657)
r.m.s. deviations				
bond lengths (Å)	0.005	0.005	0.006	0.007
bond angles (deg)	0.78	0.83	0.96	0.85
Ramachandran plot				
favored (%)	98.25	98.25	98.03	98.25
allowed (%)	1.75	1.75	1.97	1.75
outliers (%)	0.00	0.00	0.00	0.00
Clashscore	0.94	1.61	1.76	1.88
average B-factor	16.72	19.02	15.09	20.36

a Values in parentheses are for the highest resolution shell.

### Enzyme Assays

*Bo*PDA bound to a divalent metal cation was prepared using 20 mM solutions of either ZnCl_2_, NiCl_2_, CoSO_4_ or CuCl_2_. Each metal solution was added to *Bo*PDA at 0.5 mM in 1 μL increments until at least a 4-fold molar excess was achieved. Solutions were then buffer exchanged into 20 mM NaH_2_PO_4_ pH 7.0 and concentrated in 10 kDa MWCO Vivaspin 500 centrifugal concentrators (Sartorius) at 4 °C, 5000*g* using an AccuSpin Micro 17R benchtop centrifuge (Fisher Scientific). *Bo*PDA was also buffer exchanged into 20 mM NaH_2_PO_4_ pH 7.0 without any additions to create a no metal control, and concentrated as described above. Protein concentrations were measured using a Nanodrop One^C^ (Thermo Scientific), then samples were flash frozen in 20 μL aliquots and stored at −80 °C.

Two enzymatic assays were utilized to test for *Bo*PDA deacetylase activity. First, the Enzychrom Acetate Kit (Bioassay Systems) was used with the following substrates: chitin from shrimp shells (Sigma), and corn xylan (BioCore). Conditions tested were: no metal, Zn^2+^, Co^2+^ and Ni^2+^. *Bo*PDA at a final concentration of 0.1–0.3 mg/mL was incubated overnight with 20 mg of each substrate in 300 μL of 20 mM NaH_2_PO_4_ pH 7.0. The protein–substrate solution was agitated for 16 h at 37 °C in an orbital shaker or rotary mixer. Samples were then centrifuged for 5 min at 13,000 rpm in an AccuSpin Micro 17R benchtop centrifuge (Fisher Scientific) before being used for enzyme activity assays. The reaction mixtures were prepared for analysis by incubating 10 μL sample with 90 μL of the Enzychrom Acetate Kit working reagent in a clear 96-well plate (Greiner). After 30 min, fluorescence at 570 nm was detected using a BioTek Synergy HTX Microplate Reader (Agilent). A standard curve was prepared using 0.0–0.9 mM acetic acid to determine background fluorescence as well as to convert fluorescence values to acetate concentration. A linear regression was used to calculate acetate concentrations for all samples, after subtracting the blank. Reactions were performed in triplicate at room temperature.

*Bo*PDA deacetylase activity was also investigated using general substrate *p*-nitrophenyl acetate (*p*NPA). Reaction mixtures were 1 mL containing 330 μM *Bo*PDA, 20 mM NaH_2_PO_4_ pH 7.0, and substrate (*p*NPA). Conditions tested were: no metal, Zn^2+^, Co^2+^, and Ni^2+^. Metal bound *Bo*PDA was prepared as described above. *p*-Nitrophenyl acetate (*p*NPA) substrate concentrations were varied from 3.4–340 mM. Absorbance was measured at 405 nm over 30 s in cuvettes using a Genesys 10 UV–Vis spectrophotometer (Thermo Scientific). Reactions were performed in duplicate at room temperature. Blank readings were determined by substituting 20 mM NaH_2_PO_4_ pH 7.0 buffer for the enzyme.

### Carbohydrate Binding Assay

An SDS-PAGE based binding assay described previously^39^ was modified and completed with *Bo*PDA as follows. Reactions were 20 μL containing *Bo*PDA at 0.25 mg/mL, 50 mM Tris–HCl pH 7.4, 150 mM NaCl and 1 mg of substrate. Samples were mixed and incubated for 4 h at 25 °C. Carbohydrate substrates used were cellulose (Sigma), chitin from shrimp shells (Sigma), corn xylan (BioCore), and amylopectin from corn (MP Biochemicals). After incubation, the samples were centrifuged at 17,000*g* for 5 min an AccuSpin Micro 17R benchtop centrifuge (Fisher Scientific) and 5.0 μL of the supernatant was removed. An equal volume of 2× Laemmli Sample Buffer (100 mM Tris-Cl pH 6.8, 4% (w/v) sodium dodecyl sulfate, 0.2% (w/v) bromophenol blue, 20% (v/v) glycerol, and 6% β-mercaptoethanol) was added to each supernatant sample. After boiling for 10 min at 100 °C, 7 μL of each sample mixture was loaded on to a 15% gel (8.3 × 7.3 cm, 0.75 mm thickness) for analysis via Tris-Glycine-SDS-PAGE. Coomassie Brilliant blue stained gels were imaged with a ChemiDoc (Bio-Rad) and bands were quantified using ImageLab (Bio-Rad).

## Results

### Purification of *Bo*PDA

**Figure 1:**
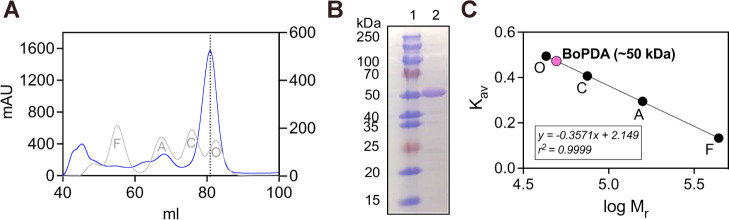
Purification of *Bo*PDA. (A) Elution profile for *Bacteroides ovatus* polysaccharide deacetylase (*Bo*PDA) (blue line) from a HiLoad 16/60 Superdex 200 pg Size Exclusion Chromatography (SEC) column. The SEC column was calibrated separately (gray line). *Bo*PDA eluted at 80.87 mLs (dotted line) from the SEC column and (B) was visualized on a 12% SDS-PAGE with Coomassie Blue. Pure *Bo*PDA ran as a single band (Lane 2) near the 50 kDa standard marker (Lane 1). (C) SEC calibration curve generated from protein standards elution profile (black circles) and with the calculated molecular weight of *Bo*PDA shown (pink circle). Protein standards used for SEC column calibration were O = ovalbumin (43 kDa), C = conalbumin (75 kDa), A = aldolase (158 kDa) and *F* = ferritin (440 kDa).

Initial sequence analysis of *B. ovatus* gene BACOVA_3992 (WP_004300358) using BlastP^40^ indicated it is a member of the carbohydrate esterase 4 (CE4) superfamily which typically uses a metal-dependent acid/base mechanism for the N- or O-deacetylation of carbohydrates.^41^*Bacteroides ovatus* polysaccharide deacetylase (*Bo*PDA) is 499 aa and a construct comprising residues 20–499 was purified (Figure 1). Sequence analysis showed that the N-terminus (residues 1–20) contains a lipoprotein signal peptide, which was excluded in the final purification construct.^42^ Signal Pro 6.0^43^ predicts a Sec/SPII signal peptide and a cleavage site between *Bo*PDA residues G^21^ and C^22^ of the lipobox (^19^MSG**C**^22^) with high probability (>99.55%). Lipid attachment occurs at the strictly conserved Cys in the +1 position. The purified protein also includes one unnatural amino acid at the N-terminus (G) remaining after cleavage of the polyhistidine tag.

Based on the sequence, ProtParam^44^ predicted a monomeric weight for the purified protein of 55.5 kDa. The elution volume (*V*_e_) of *Bo*PDA from a HiLoad 16/60 Superdex 200 pg size exclusion column (SEC) was 80.87 mL (Figure 1A; blue line). Consistent with the predicted monomer size SEC-purified BoPDA migrated as a single band (Lane 2) with a molecular weight near the 50 kDa standard marker (Lane 1, kDa) on a 12% SDS-PAGE stained with Coomassie Blue (Figure 1B). To estimate the oligomeric state in solution, separate calibration of the SEC column was carried out to generate a standard curve by plotting *K*_av_ (partition coefficient calculated using *V*_e_) vs log relative molecular weight (*M*_r_) of the protein standards (Figure 1A, C; gray line). The elution volume of *Bo*PDA fell between that of standard proteins conalbumin (C, 75 kDa) and ovalbumin (O, 43 kDa), with its *M*_r_ calculated as 49.4 kDa (Figure 1C; pink circle) indicating a monomeric form in solution. Most CE4 deacetylases are monomers, however some have been shown to oligomerize.^41,45^

### Crystal Structure Overview

The *Bo*PDA crystal structures revealed a unique nonmodular domain architecture not previously observed in CE4 enzymes.^41^ Four crystal structures of *Bo*PDA bound to divalent cations were solved: to Co^2+^, Ni^2+^, Zn^2+^, and Cu^2+^ at 1.42 Å, 1.48 Å, 1.36 Å and 1.56 Å respectively (Table 1). Co^2+^, Ni^2+^ and Zn^2+^ are the most common cations used by CE4 enzymes for catalysis.^41^ We also included Cu^2+^, a cation that was unlikely to support CE4 family enzymatic activity, to compare cation binding promiscuity by *Bo*PDA. Another CE4 enzyme was reported previously to have crystallized bound to Cd^2+^, which does not typically support CE4 enzyme activity, however that refined structure is not available for comparison.^46^ In agreement with the SEC data, *Bo*PDA crystallized as a monomer, further supported by PDBePISA analysis of the protein interfaces which did not predict any quaternary structure.^47^

**Figure 2:**
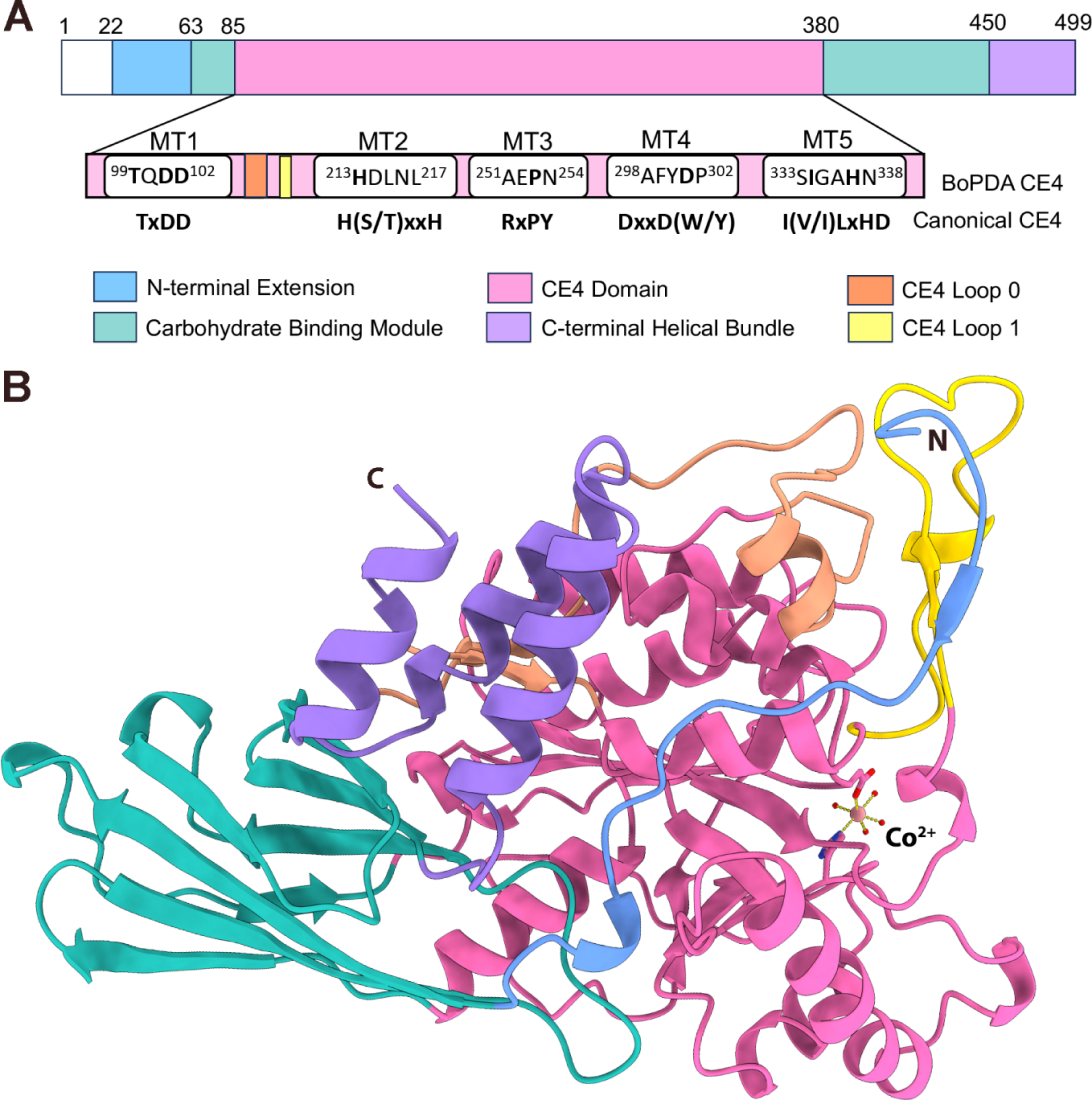
Structural overview of *Bo*PDA. (A) Domain architecture of *Bo*PDA showing a catalytic carbohydrate esterase superfamily 4 (CE4) domain (pink), a carbohydrate binding domain (CBM; teal), a N-terminal extension (blue) and C-terminal helical bundle (purple). Two loop regions important for substrate specificity in the CE4 domain are also highlighted (orange and yellow). The CE4 domain inset shows conserved residues in the five canonical motifs (MT1-MT5) and the corresponding residues in *Bo*PDA. The N-terminal (white, residues 1–22) is a predicted signal peptide. (B) Crystal structure of *Bo*PDA bound to Co^2+^ (light pink, sphere) with residues His^213^ and Asp^102^ from the active site shown as sticks, and water molecules as red spheres. Bonds to the metal cation are shown in yellow. The domains are colored as in (A).

The *Bo*PDA structure contains a CE4 domain (also called the NodB homology domain) (pink, Figure 2), a β-sandwich domain (carbohydrate binding module) (teal, Figure 2), and additional extensions on the N- and C-termini (Figure 2). The unusual nonmodular organization arises due to a domain insertion which occurs when one domain (insert) is inserted into another domain (parent).^5^ In *Bo*PDA the β-sandwich domain (parent) is interrupted sequentially by the CE4 domain. Residues 86–380 constitute the CE4 domain, while residues 63–85 then 381–441 fold into a singular β-sandwich domain flanked on either termini by short extensions (Figure 2). Domain insertions are uncommon, as only ∼9% of proteins are known to contain a domain insertion.^48^ The final protein models did not include residues 20–40 due to disorder. The N-terminal extension that we were able to model (residues 40–62; blue, Figures 2, 5A) is likely part of a linker given the lipid attachment site at residue 22. The predicted signal peptide (residues 1–21) is shown in white (Figure 2A). Following the β-sandwich domain is a three helix α-helical bundle at the C-terminus (residues 451–499; purple, Figure 2).

**Figure 3:**
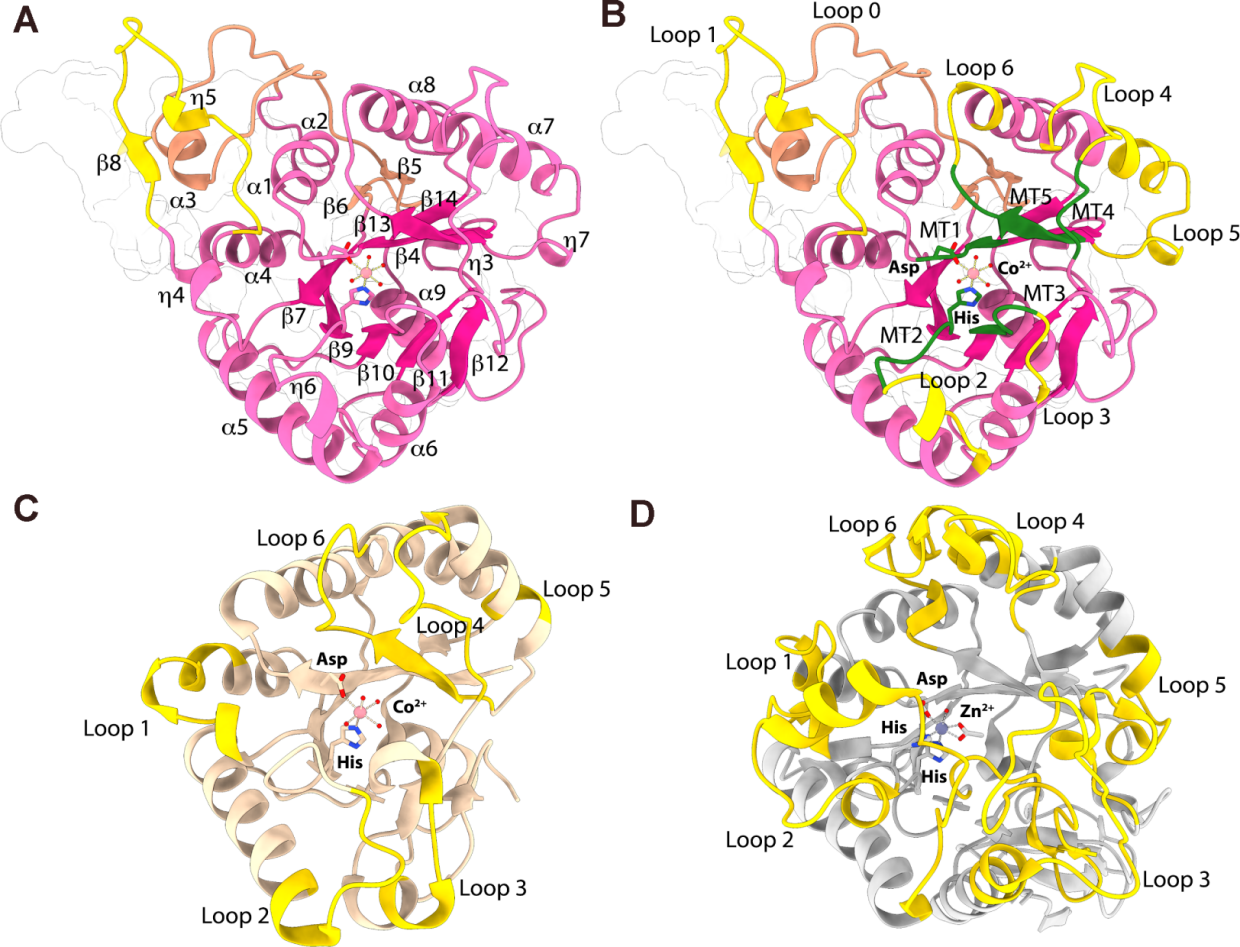
CE4 domain of *Bo*PDA. (A) Annotated secondary-structure elements α-helices (α), β-sheets (β), and 3_10_-helices (η) of the CE4 domain. The protein is rotated 90° relative to Figure 2A, with the other domains grayed out for clarity. Within the CE4 domain the α-helices (α) are colored pink and β-sheets are colored dark pink, whereas Loops 0 and 1 are colored orange and yellow respectively as in Figure 2. The CE4 domain adopts a deformed (β/α)_8_ barrel fold, with the active site located near the center of the β-barrel. Active site residues His^213^ and Asp^101^ are shown as sticks, the Co^2+^ ion as a light pink sphere, water molecules as red spheres, and bonds to the metal as yellow dotted lines. (B) CE4 motifs (MT1-MT5) are shown in green. Known CE4 substrate specificity Loops 1–6 are shown in yellow, while Loop 0 which is novel to *Bo*PDA is shown in orange. Other secondary structure elements are colored as in (A). (C), (D) Active site (sticks) and substrate specificity Loops 1–6 (yellow) in (C) *Clostridium thermocellum* acetylxylan esterase (*Ct*AxeA)(tan, PDB 2c79) and (D) *Vibrio cholerae* chitin deacetylase (*Vc*CDA) (white; PDB 4ny2). Only the CE4 domains of each protein are shown. *Ct*AxeA has a Co^2+^ ion bound (pale pink; sphere) and bonds to the metal are shown in yellow. *Vc*CDA has a Zn^2+^ (gray; sphere) and acetate ion (white; sticks) bound, and bonds to the metal are shown in gray.

**Figure 4:**
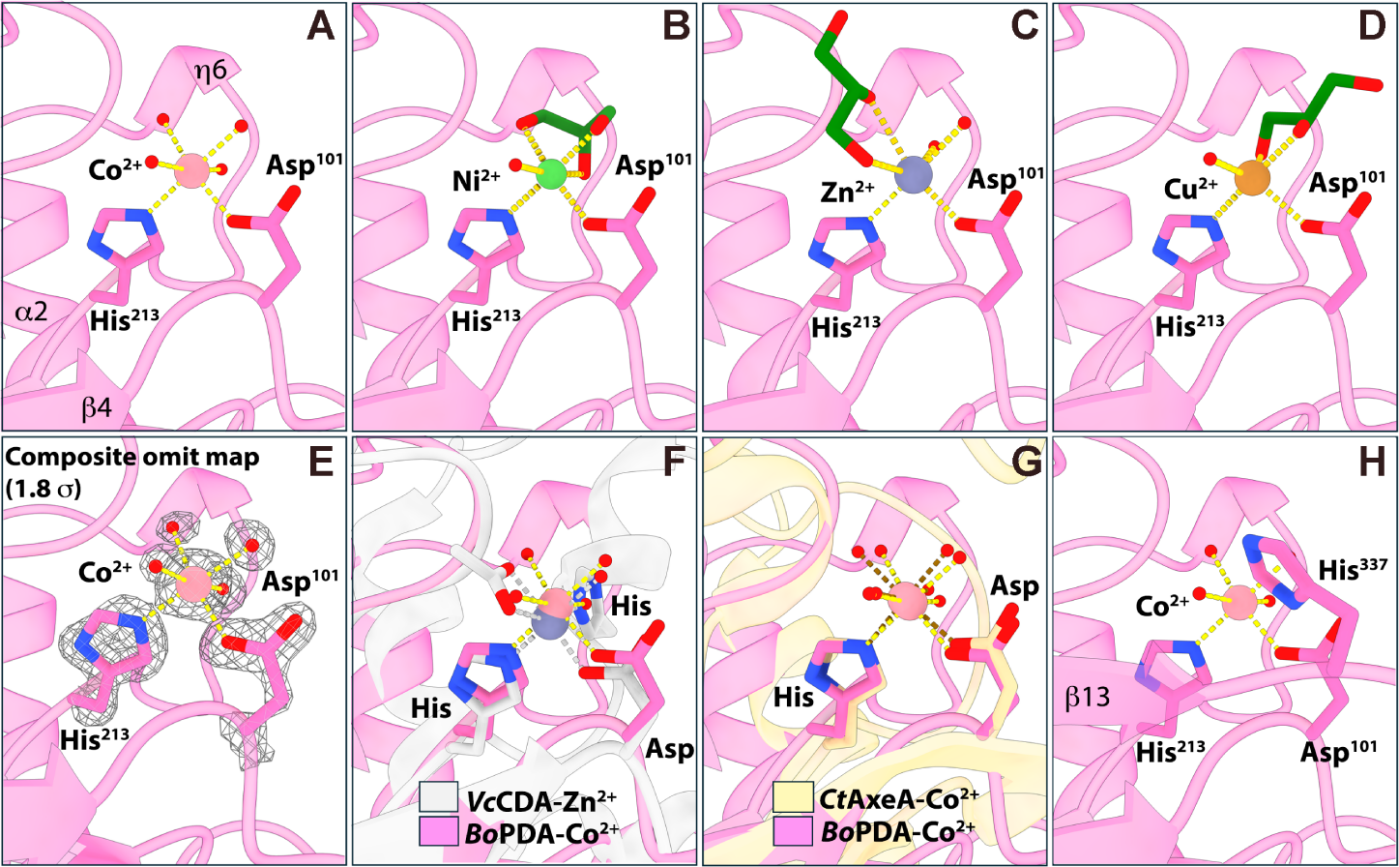
Metal binding in *Bo*PDA active site. *Bo*PDA residues His^213^ and Asp^101^ (pink, sticks) coordinating (A) Co^2+^ (pale pink; sphere), (B) Ni^2+^ (light green, sphere), (C) Zn^2+^ (gray; sphere) and (D) Cu^2+^ (brown, sphere) ions. Water molecules are shown as red spheres and glycerol molecules as dark green sticks. Bonds to the metal ion are shown in yellow. (E) Composite omit map contoured to 1.8σ showing active site of *Bo*PDA- Co^2+^ structure. (F) Overlay of *Bo*PDA- Co^2+^ with *Vibrio cholerae* chitin deacetylase (*Vc*CDA, PDB 4ny2) showing its canonical CE4 domain His-His-Asp motif binding a Zn^2+^ ion (gray; sphere), with waters and an acetate ion (white; sticks) completing the coordination. Bonds to the Zn^2+^ are shown in gray. (G) Overlay of *Bo*PDA- Co^2+^ with *Clostridium thermocellum* acetylxylan esterase (*Ct*AxeA, PDB 2c79) showing a similar His-Asp motif coordinating a Co^2+^ ion (pale pink; sphere). Bonds to the *Ct*AxeA bound Co^2+^ are shown in brown. (H) Orientation of MT5 catalytic His^337^ in active site of the *Bo*PDA- Co^2+^ structure.

**Figure 5:**
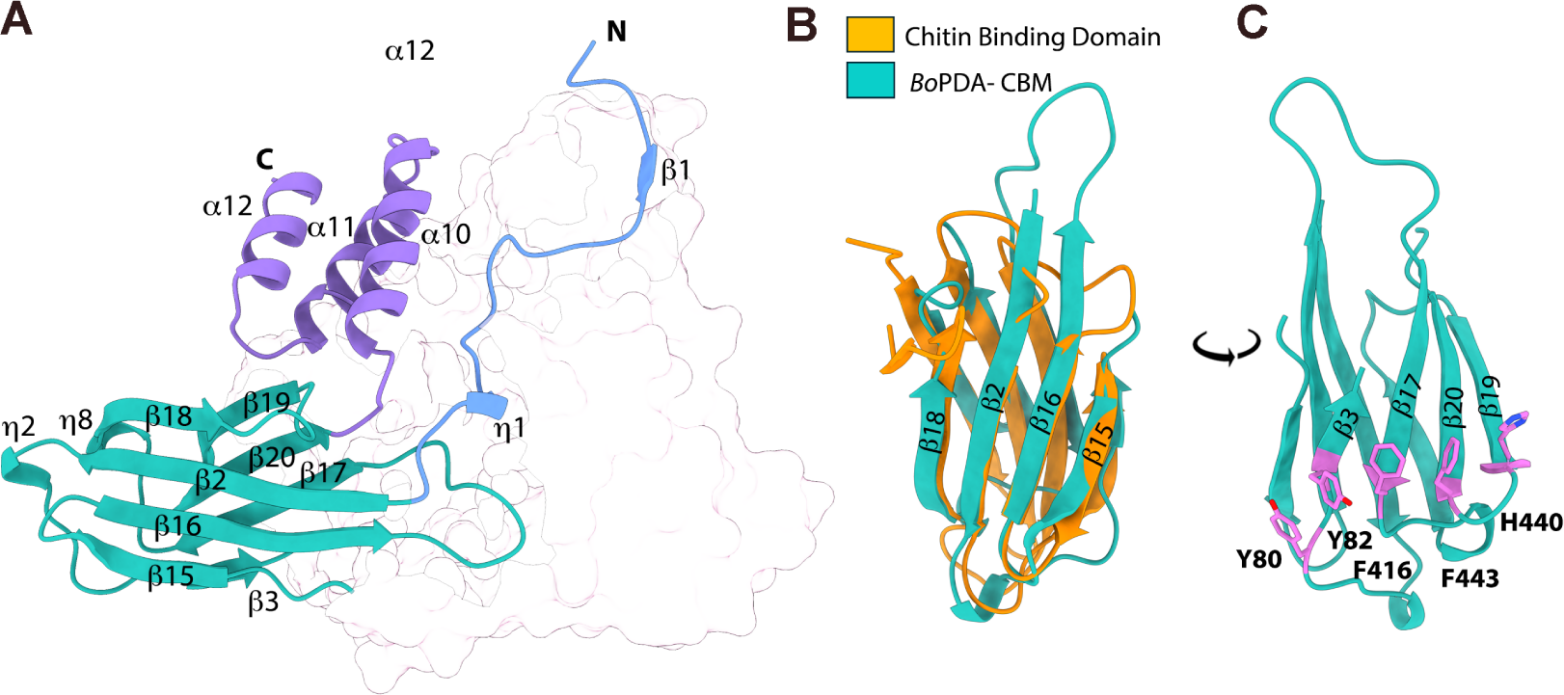
Carbohydrate binding module and other domains. (A) Annotated secondary-structure elements α-helices (α), β-sheets (β), and 3_10_-helices (η) of the carbohydrate binding module (CBM, teal), N-terminal extension (blue) and C-terminal helical bundle (purple), with the CE4 domain grayed out for clarity. (B) Structural alignment of the *Bo*PDA CBM with the chitin binding domain of *Pyrococcus furiosus* chitinase (PDB 2cwr, r.m.s.d. 1.01 Å). (C) Aromatic residues in the *Bo*PDA CBM that form the predicted carbohydrate binding platform are shown as sticks (violet).

**Figure 6:**
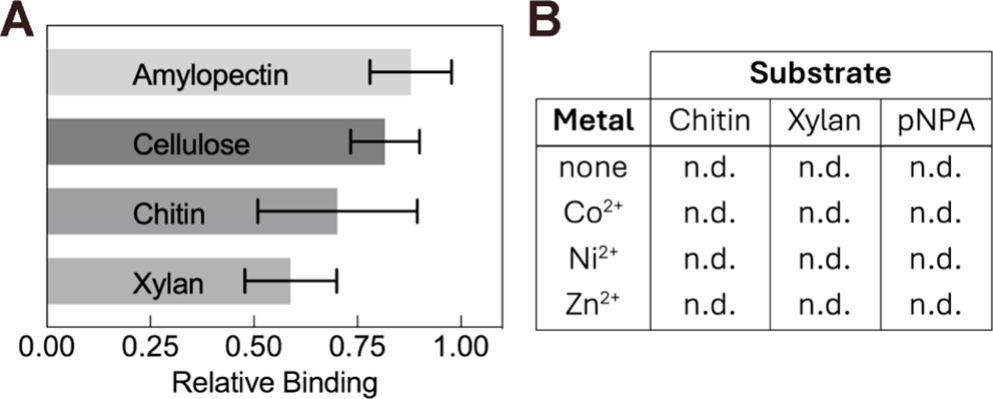
*Bo*PDA substrate binding and activity assays. (A) Binding assay of *Bo*PDA to common carbohydrates. SDS-PAGE bands were quantified, and the histogram shows densities relative to a protein only control lane. The lower relative binding values for chitin and xylan indicate carbohydrate was bound. (B) *Bo*PDA showed no detectable enzymatic activity (n.d.) in any combination of substrate and metal shown in table. EnzymChrom Acetate Kit (Bioassay Systems) used with all substrates except *p*-nitrophenyl acetate (*p*NPA). *p*NPA was used in a spectrophotometric assay.

### CE4 Domain

**Figure 7:**
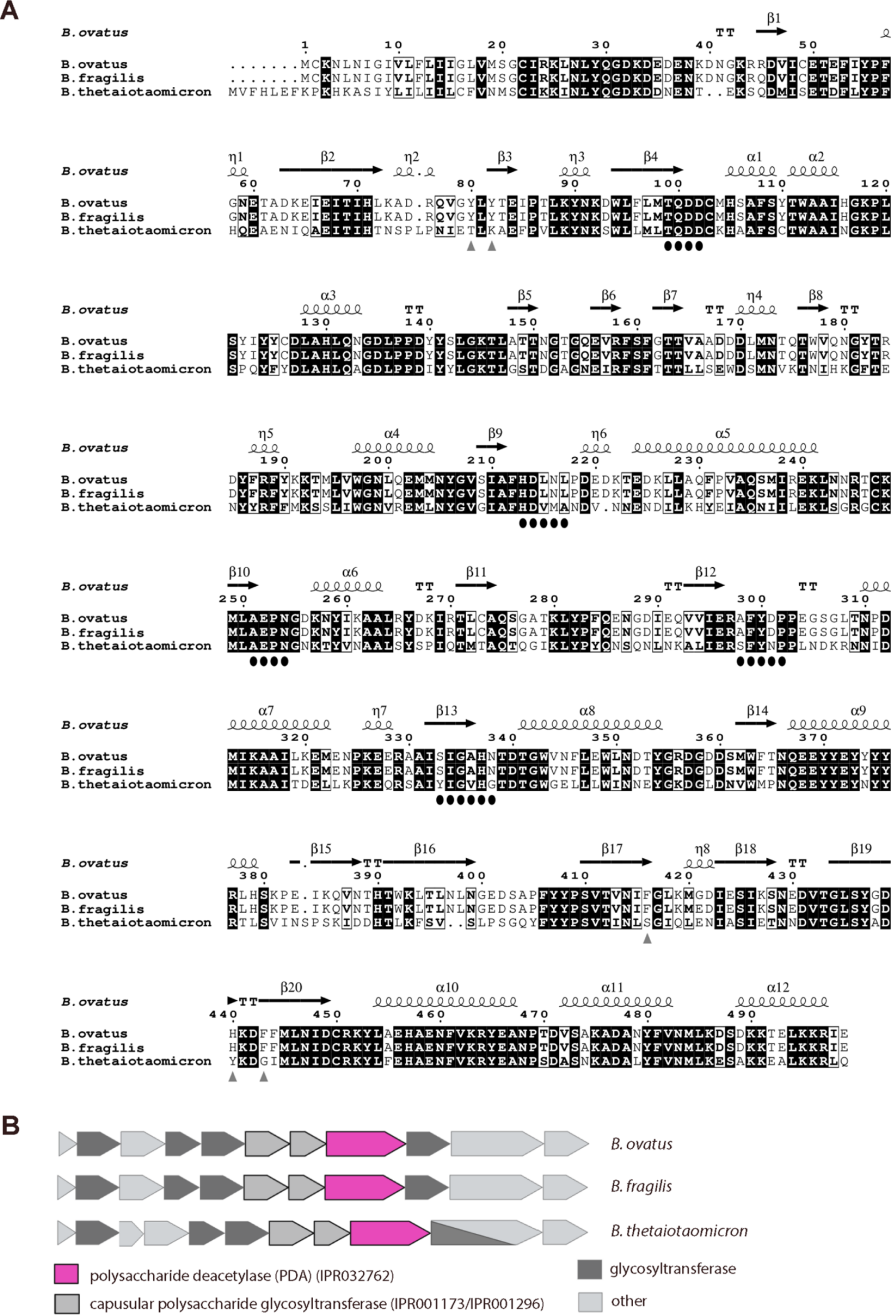
Sequence alignment and genomic neighborhood of *Bo*PDA. (A) Primary-sequence alignment of *B. ovatus* polysaccharide deacetylase (*Bo*PDA) with homologues from *B. fragilis* and *B. thetaiotaomicron*. Secondary-structure elements of *Bo*PDA are shown: α-helices (α), β-sheets (β), 3_10_-helices (η) and β-turns (TT). Identical residues are shown in white on a black background, while conserved residues are shown in bold and related residues are boxed. Black ovals indicate conserved CE4 motifs (MT1-MT5). Residues predicted to be part of the carbohydrate binding platform in the CBM are indicated by gray triangles. The image was generated using ESPript.^69^ (B) Genome neighborhoods for polysaccharide deacetylases (pink; IPR0332762) from *B. ovatus*, *B. fragilis* and *B. thetaiotaomicron*. Integrated Microbial Genomes and Microbiomes (IMG/M)^67^ was used to obtain genomic neighborhoods.

The 294 aa *Bo*PDA CE4 domain adopts a characteristic distorted (β/α)_8_ barrel fold with 8 parallel β-strands (β4, β7, β9- β14), surrounded by 8 α-helices (α1, α2, α4−α9) which is similar to several other CE4 family members (Figure 3A).^46,49,50^ Five conserved motifs (MT1-MT5; Figures 2 and S1) typify the catalytic CE4 domain.^41^ The motifs are clustered around the β-barrel forming the active site and substrate binding pocket, with MT1 in β4, MT2 between β9 and η6, MT3 in β10 and loop following, MT4 between β12 and α7, and MT5 in β13 (Figures 3A, B and 7A). Substrate specificity of CE4 enzymes is determined by six loops (Loop 1–6) found between the conserved motifs. Loop 1 is found between MT1 and MT2, Loop 2 between MT2 and MT3, Loop 4 and 5 are both between MT3 and MT4, and Loop 6 is found after MT5 (Figure 3B).^41^

Motif 1 and 2 contain residues that form the canonical His-His-Asp metal-binding triad in the active site. Motif 1 is typically four residues, TFDD, with the first aspartic acid acting as a general base during catalysis while the second participates in metal coordination. Two conserved histidines from Motif 2 (H(S/T)xxH) complete the canonical His-His-Asp triad. In *Bo*PDA, Motif 1 is mostly conserved (^100^TQDD^102^), however the second histidine of MT2 is absent (^213^HDLN^217^) (Figure 2A).

The remaining motifs, MT3-MT5, in the *Bo*PDA CE4 domain show more divergence from the canonical sequences (Figure 2A). To map the location of these motifs we used structural alignments of the *Bo*PDA structure with previously characterized CE4 enzymes *Vibrio cholerae* chitin deacetylase (*Vc*CDA; PDB 4ny2) and *E. coli* poly-β-1,6-GlcNAc deacetylase (*Ec*PgaB; PDB 3vus) (Figure S2).^51,52^ The variable loop lengths for Loops 1–6 in the CE4 domain makes it difficult to easily generate multiple sequence alignments between most CE4 family members,^40^ and this was exacerbated in *Bo*PDA by the nonmodular domain architecture. MT3 (RxPY) and MT4 (DxxD(W/Y) create two sides of the substrate binding groove, however only one residue each in *Bo*PDA is conserved (^251^AEPN^254^ and ^298^AFYDP^302^ respectively) (Figure S1). Alignment of *Bo*PDA-Co^2+^ with a structure of *Vc*CDA bound to triacetylchitotriose (PDB 4oui) demonstrate where the *Bo*PDA substrate is likely to be positioned close to the conserved motifs (MT1-MT5) (Figure S1B). The conserved arginine in MT3, which is absent in *Bo*PDA, is actually found closer to MT4 in poly-β-1,6-GlcNAc deacetylases.^41,49^ Alignment of *Bo*PDA with the poly-β-1,6-GlcNAc deacetylase *Ec*PgaB finds that *Bo*PDA has an arginine (Arg^297^) in that later position as well (Figure S1). MT5 (I(V/I)LxHD) usually contains a leucine that forms a section of the hydrophobic pocket which accommodates part of the substrate, however it is replaced by the smaller hydrophobic residue alanine in *Bo*PDA (^333^SIGAHN^338^). In addition, the histidine residue which acts as the general acid during catalysis is found in MT5. Significantly, this catalytic histidine (His^337^) is preserved in MT5, indicating *Bo*PDA is likely enzymatically active (Figures S1, S3). CE4 enzymes that lack the MT5 histidine are catalytically inactive even if they retain the His-His-Asp triad.^41^ There is only one other example of a CE4 enzyme that lacks a conserved MT2 metal binding histidine but retains the MT5 catalytic histidine, *Clostridium thermocellum* AxeA (*Ct*AxeA), and it has been shown to be active against acetylxylan (Figures 3C, S2, and S3).^46^

Typically, the core (β/α)_8_ barrel fold of CE4 domains is ∼150–200 residues. Some CE4 domains are larger with differences in size mostly localized to the six loop regions (Loop 1–6) found between the conserved motifs (MT1-MT5). For example, the CE4 domain of *Vibrio cholerae* chitin deacetylase (*Vc*CDA) is 312 aa because many of its loops are long (>20 aa), while in contrast all the loops in the 208 aa *Ct*AxeA CE4 domain are short (<14 aa),^41,46,51^ In the 294 aa *Bo*PDA CE4 domain, while Loops 2–6 are short (5–15 aa), Loop 1 is long (20 aa) (Figure 3B).

Additionally in *Bo*PDA there is a long loop (38 aa) between α2 and β7 that is not observed in any other CE4 enzymes we examined (residues 121–159; orange, Figure 3B). As this insertion occurs before Loop 1, we have termed it Loop 0 (Figure 3B). The length, composition and spatial position of the Loops 1–6 have been hypothesized to determine substrate specificity.^41,51^ The active sites of *Bo*PDA and *Ct*AxeA are open (Figure 3B,C), whereas in *Vc*CDA, the long loops cluster in front of the β-barrel constricting access to the substrate binding and active sites (Figures 3D and S2).

Interestingly in *Bo*PDA Loops 0 and 1 are off to one side of the (β/α)_8_ barrel with a hydrogen bond network formed between five Loop 0 residues (Tyr^125^, Asp^127^, His^130^, Asn^133^, Asp^135^) and six Loop 1 residues (Thr^176^, Trp^177^, Asn^180^, Arg^188^, Phe^189^, Lys^192^). Adjacent to Loops 0 and 1 is the C-terminal α-helical bundle (residues 451–499; purple, Figures 2B and 5A). According to Dali,^53,54^ the C-terminal α-helical bundle looks structurally most similar to the C3-binding domain of Efb (Efb-C) from *Staphylococcus aureus* (PDB 2gox) with r.m.s.d. 0.9 Å, but all of the helices in Efb-C are slightly longer than in *Bo*PDA.^55^ Functionally Efb-C mediates a protein–protein interaction along its second helix, and the analogous helical face responsible is accessible in *Bo*PDA, so could possibly have a similar function. However, given the arrangement of the C-terminal α-helical bundle along with both Loop 0 and Loop 1, it seems more likely they may form a surface that contributes to substrate specificity in *Bo*PDA.

Our four metal-bound *Bo*PDA crystal structures allowed us to investigate the metal-binding strategy of *Bo*PDA given it lacks the canonical His-His-Asp triad. In the Co^2+^ bound *Bo*PDA structure the divalent cation is coordinated with a His^213^-Asp^102^ dyad and four water molecules (Figure 4A). The Co^2+^ ion is coordinated through typical octahedral geometry when validated using the *CheckMyMetal* (CMM) server, with acceptable values for all the CMM parameters.^56−59^ A composite omit map was generated for the *Bo*PDA-Co^2+^ structure and it shows clear, defined density for the Co^2+^ cation and each water molecule modeled (Figure 4E).

In the Ni^2+^, Zn^2+^ and Cu^2+^ bound structures, the metal ions are also coordinated by the His^213^-Asp^102^ dyad however unlike Co^2+^ they were not able to be modeled at full occupancy (Figure 4 B,C,D). Crystallographic estimations of the Ni^2+^, Zn^2+^ or Cu^2+^ ion’s occupancy after multiple rounds of refinement suggest occupancies of ∼90% for Ni^2+^, ∼ 20% for Zn^2+^ and ∼75% for Cu^2+^. Additionally, instead of four water molecules completing the metal binding site as in the *Bo*PDA-Co^2+^ structure, there is a glycerol molecule (green, sticks) and 1 to 2 waters interacting with the Ni^2+^, Zn^2+^ or Cu^2+^ ion (Figure 4B, D). Composite omit maps for the *Bo*PDA-Ni^2+^, -Zn^2+^ and -Cu^2+^ structures showed clear density supporting the inclusion for all modeled molecules, including glycerol (Figure S4). Despite the low occupancy of the Zn^2+^, when a water is modeled in its place there is a strong positive peak (green) in the F_o_-F_c_ difference map indicating it is not a good fit. This is resolved when Zn^2+^ is included and its occupancy is refined. Validation with the CMM server for these structures (Ni^2+^, Zn^2+^ or Cu^2+^) revealed that many CMM parameters, like the geometry and valence, were not in the acceptable range. Given our high resolution for the *Bo*PDA-Ni^2+^, *Bo*PDA-Zn^2+^ and *Bo*PDA- Cu^2+^ structures (1.42 Å, 1.36 Å, and 1.56 Å respectively), and that none of the cations refine to complete occupancy, these metals may not be tightly bound. Taken together, this suggests that the preferred metal for *Bo*PDA is likely Co^2+^, and it may have reduced or no activity with Ni^2+^, Zn^2+^, or Cu^2+^.

The *Bo*PDA active site is arranged similarly to other CE4 enzymes. Overlays of the active site of the *Vc*CDA- Zn^2+^ structure shows that the MT2 metal binding histidine is replaced by a water molecule in *Bo*PDA (Figure 4F). *Bo*PDA is now the second case of a CE4 enzyme using a dyad for metal coordination, the only other example being *Ct*AxeA (Figures 3C and 4G).^46^ The MT4 catalytic histidine (His^337^) in *Bo*PDA is positioned as in other CE4 family members, on the other side of the active site (Figures 4H and S3).^41^ Compared to an apo-*Bo*PDA structure (PDB 4dwe, unpublished, Joint Center for Structural Genomics), there is no significant conformational change to the *Bo*PDA overall structure upon metal binding, with an r.m.s.d. of 0.113 Å, 0.141 Å, 0.102 Å and 0.102 Å for Co^2+^, Ni^2+^, Zn^2+^ and Cu^2+^ bound structures respectively to apo-*Bo*PDA. Local rearrangements at the active site accommodate the metal cation, which when bound displaces a water molecule that occupied the apo-*Bo*PDA active site.

### Carbohydrate Binding Module

*Bo*PDA has an 84 aa β-sandwich domain (residues 63–85 and 381–441) that folds into two 4-stranded antiparallel β-sheets (β15-β16-β2-β18 and β3-β17-β20-β19) with two 3_10_-helices (η7 and η8)(Figure 5A). Residues 63–85 form β2, η7, and β3, which is followed sequentially by the CE4 domain. Residues 381–441 form the remaining β-strands and 3_10_-helix. Structural homologues of *Bo*PDA were found with Dali,^53,54^ however the top matches were proteins with a CE4 domain. Searching Dali with only the β-sandwich domain coordinates returned results specific to the domain. Chitin binding domains such as from archaeal chitinase (PDB 5xsv) were top hits suggesting it is a carbohydrate binding module (CBM). Alignment with the chitin binding domain of *Pyrococcus furiosus* chitinase (PDB 2cwr, r.m.s.d. 1.01 Å) shows the high structural similarity (Figure 5B).

Carbohydrate binding modules (CBM) are noncatalytic domains found widely in eukaryotes, bacteria, archaea and viruses.^60,61^ Many carbohydrate active enzymes (CAZymes) have one or more CBMs modularly arranged with their catalytic domains.^61^ Functionally, CBMs can increase the catalytic efficiency and substrate specificity of the CAZyme, and have also been shown to influence the overall thermodynamic properties.^61^ CBMs can be classified into 7 fold families and 3 types based on ligand binding pattern, then further sorted into one of 99 sequence families.^60,61^ CBM fold families include the β-sandwich, β-trefoil, and OB (oligonucleotide/oligosaccharide binding) fold.^60^ The CBM ligand binding patterns are Type A, which typically have a flat binding surface, Type B, which has a shallow groove for binding glycans, and Type C, which typically binds only small saccharides or glycan termini.^60,61^

The CBM in *Bo*PDA falls into Fold Family 1, the β-sandwich. Fold family 1 is the most common CBM fold.^60,61^ The CBM β-sandwich fold is typically comprised of two stacked β-sheets, each with 3–6 antiparallel β-strands, with the carbohydrate binding site often located along one face.^60,61^ Sequence and structural analysis of the *Bo*PDA-CBM reveal a potential binding site platform on the β3-β17-β20-β19-sheet face of the β-sandwich (Figure 5C). Aromatic residues Tyr^80^, Tyr^82^, Phe^416^, His^440^ and Phe^443^ form a “twisted” platform in a shallow groove, where the planes of each amino acid is rotated relative to each other (Figures 5C and 7A). The “twisted” platform is one of three distinct hydrophobic platforms in CBM-binding sites, with the other two being flat “planar”, or “sandwich” platforms.^60,61^ Identification of this binding site suggests classification of the *Bo*PDA-CBM as a Type B CBM, in the CBM2b sequence family.

### Carbohydrate Binding and Deacetylase Activity

Relative binding assays with *Bo*PDA against the common polysaccharides cellulose, amylopectin, chitin and xylan were performed to gain preliminary insight into substrate preference. *Bo*PDA did not bind cellulose or amylopectin appreciably, and bound xylan and chitin moderately well (Figures 6A, S5, and Table S1). High substrate preference would be indicated by relative binding values of <0.25, as has been seen previously between the substrate chitin and chitin binding domains.^39^ Both amylopectin and cellulose are polymers of glucose, while chitin is composed of N-acetylglucosamine (GlcNAc) and xylan is mostly xylose. As mentioned previously, *Bo*PDA has a conserved arginine in an identical position closer to MT4 as found in poly-β-1,6-GlcNAc deacetylases.^41,49^ Together, this qualitatively suggests that the natural substrate of *Bo*PDA may have GlcNAc and/or xylose components. As *Bo*PDA binds substrates chitin and xylan moderately, *Bo*PDA likely has high specificity for a yet unknown polysaccharide substrate. Future carbohydrate binding studies of *Bo*PDA could employ a wider variety of substrates that have been used successfully with other CE4 enzymes, for example shorter chitooligosaccharides were utilized in studies of *Vibrio cholerae* chitin deacetylase (*Vc*CDA).^51^ In addition, to obtain quantitative binding data methods such as isothermal titration calorimetry can be employed.

*Bo*PDA deacetylase activity using substrates chitin and xylan was evaluated. Three divalent metal cofactors were tested (Co^2+^, Ni^2+^, and Zn^2+^) with each substrate. No activity was detected in any combination of substrate and metal cofactor (Figure 6B and Table S2). Given our crystal structures we expected to see high activity with Co^2+^, and partial/no activity with Ni^2+^ and Zn^2+^, however we saw no activity with any substrate tested. While the acetate assay we chose had been used successfully to evaluate another CE4 deacetylase, *Bjerkandera adusta* carbohydrate esterase (*Baces*I) with the same substrates,^62^ other CE4 enzymes such as the acetyl xylan esterases from *Clostridium thermocellum* (*Ct*AxeA) and *Streptomyces lividans* (*Sl*AxeA) required development of a custom assay to measure activity.^46^ Additional evaluation of *Bo*PDA deacetylase activity was carried out using the general esterase substrate *p*-nitrophenyl acetate (*p*NPA) and the same three divalent metal cofactors, but no activity against *p*NPA was detected (Figure 6B and Table S3). Though many carbohydrate esterases are active against *p*NPA, *Ct*AxeA and *Sl*AxeA also showed no activity.^46,63^ An alternate general esterase substrate that could be used in the future is 4-methylumbelliferyl acetate, as CE4 deacetylase *Vc*CDA showed activity against it in previous studies.^51^ Further, a novel assay to evaluate *Bo*PDA enzyme activity could be developed, similar to the enzyme-coupled assay used with *Ct*AxeA and *Sl*AxeA which incorporates a custom *p*-nitrophenyl tagged acetylated xylopyranoside and β-xylosidase.^46,64^

### Genomic Context

*Bo*PDA belongs to the *Putative polysaccharide deacetylase* family (Pfam15421/IPR032762),^65^ a bacterial protein family whose members are mainly from Bacteroides. Previous research identified a group of 185 proteins specific to the Bacteroides genus and found in no others, including closely related Firmicutes and Cholrabi.^66^ In that group was *B. thetaiotaomicron* gene BT_1182, which encodes a homologous protein of 497 aa that is 98% identical to *Bo*PDA (Figure 7A). As it is a unique Bacteroides protein, the *Bo*PDA protein sequence only aligns well with closely related homologues from Bacteroides (Figure 7A). *Bo*PDA is the first structure of this unique protein, and thus the first solved in the *Putative polysaccharide deacetylase* family (Pfam15421/IPR032762).

The *Bo*PDA genome neighborhood was examined using Integrated Microbial Genomes and Microbiomes (IMG/M).^67^ We found all Bacteroides species that harbored a homologous PDA gene only contain one copy, and that the genome neighborhood is highly preserved (Figure 7B). Eleven proteins including PDA are encoded in the locus (Figure 7B). The Carbohydrate-Active enZYmes Database (CaZY, http://www.cazy.org/)^68^ does not predict this to be a polysaccharide utilization locus (PUL) therefore, we sought to predict the function of this locus through analyzing predicted protein families of genes in the locus. The *Bo*PDA locus contains two signaling proteins, a putative DNA binding protein with a predicted helix-turn-helix domain that may be a transcriptional regulator, a polysaccharide biosynthesis membrane transport protein, and six glycosyltransferases. Significantly, two glycosyltransferases are from the protein families Pfam00535/IPR001173 and Pfam00534/IPR001296. These glycosyltransferases are involved in capsular polysaccharide (CPS) biosynthesis, and were used in a recent study to help define CPS loci in *B. thetaiotaomicron*.^27^ Previously characterized CPS loci in Bacteroides contain a combination of signaling proteins, transcriptional regulators, glycosyltransferases, and membrane transport proteins.^25,27,70,71^ Deacetylases have also been identified in other CPS loci, such as in the polysaccharide C biosynthetic gene cluster from *B. fragilis*.^25,70^ Given the genomic context of *Bo*PDA, its natural substrate may be an unknown *B. ovatus* capsular polysaccharide and explain why we found no enzymatic activity with common carbohydrates. Thus, future functional studies of *Bo*PDA could utilize purified *B. ovatus* capsular polysaccharides as a substrate.

## Conclusion

In this study, we report the metal-bound crystal structures of *Bacteroides ovatus* polysaccharide deacetylase (*Bo*PDA). Through analysis of our structures, we hypothesize that Co^2+^ might be the catalytically relevant divalent ion for *Bo*PDA enzymatic activity. In addition, they revealed that *Bo*PDA has a distinct nonmodular structure with a domain insertion of a CE4 domain into a carbohydrate binding module (CBM) β-sandwich domain. *Bo*PDA is a lipoprotein unique to Bacteroides and likely has a role in the biosynthesis of capsular polysaccharides. As Bacteroides are a major player in the human gut microbiome, continued characterization of these basic biosynthetic processes are crucial to both increasing our understanding of microbiome-host interactions as well as to the development of new therapeutics.

## Supporting Information Available

Additional structural figures and experimental details of binding and enzymatic assays, including SDS-PAGE images and data tables (PDF)
