# Supplementary material for: Carbohydrate Deacetylase Unique to Gut Microbe Bacteroides Reveals Atypical Structure
Source: Biochemistry. 2024 Dec 12;64(1):180–91. doi: 10.1021/acs.biochem.4c00519 (PMC11713874; doi:10.1021/acs.biochem.4c00519)
Supplement: Supplementary file 1 — bi4c00519_si_001.pdf [file bi4c00519_si_001.pdf]

Supporting Information

Carbohydrate Deacetylase Unique to Gut Microbe Bacteroides Reveals Atypical Structure

Lilith A. Schwartz[1], Jordan O. Norman[2], Sharika Hasan[2], Olive E. Adamek[2], Elisa Dzuong[1], Jasmine C. Lowenstein[1], Olivia G. Yost[2], Banumathi Sankaran[3], Krystle J. McLaughlin[1,2]*

[1] *Department of Chemistry, Vassar College, 124 Raymond Ave, Poughkeepsie, NY 12604, United States*
[2] *Biochemistry Program, Vassar College, 124 Raymond Ave, Poughkeepsie, NY 12604, United States*
[3] *Advanced Light Source, Lawrence Berkely National Lab, Berkeley, CA 94720, United States*
*Email: kmclaughlin@vassar.edu

List of Supplemental Figures and Tables:

Figure S1. Conserved Motifs (MT 1-5) in the CE4 Domain of *Bo*PDA.
Figure S2. Overlays of *Bo*PDA with other CE4 Enzymes.
Figure S3. Active Site Overlays of CE4 Enzymes.
Figure S4. Active Site Composite Omit Maps.
Figure S5. Carbohydrate Binding Assay SDS-PAGE.
Table S1. Relative Binding Data for Carbohydrate Binding Assay SDS-PAGE.
Table S2: *Bo*PDA deacetylase activity with Enzychrom™ Acetate Kit
Table S3. *Bo*PDA deacetylase activity with *p*-nitrophenyl acetate (*p*NPA)

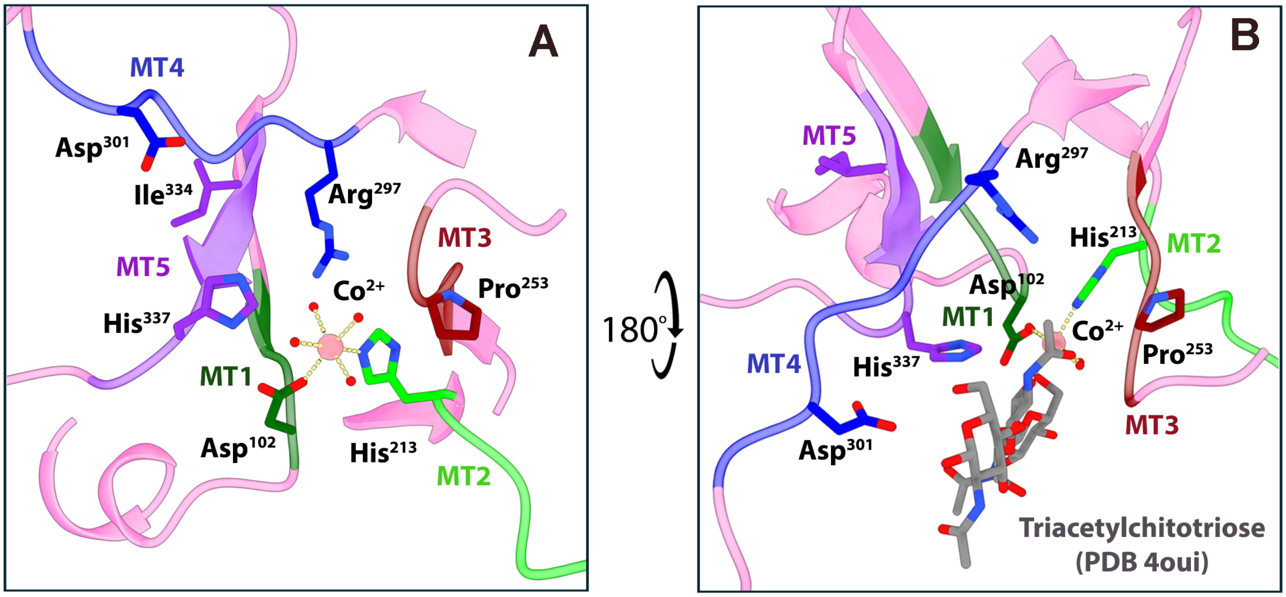

Figure S1. Conserved Motifs (MT 1-5) in the CE4 Domain of *Bo*PDA. (A) The residue regions encompassing each conserved motif (as in Fig. 2A) are colored as follows: dark green (MT1), lime green (MT2), brown (MT3), blue (MT4) and purple (MT5). Other parts of the CE4 domain are colored pink. Residues shown as sticks are the significant conserved amino acids in each motif, highlights in bold in Fig. 2A. The conserved arginine absent from the MT3 of *Bo*PDA, is found closer to MT4 in poly-β-1,6-GlcNAc deacetylases, and *Bo*PDA has an arginine in that position, shown here (Arg 297) as part of MT4. Residues from MT1 and MT 2 coordinate a Co^{2+} cation (pale pink sphere). (B) Superposition showing a bound substrate (triacetylchitoriose; gray) from the *Vibrio cholerae* chitin deacetylase (*Vc*CDA) structure (PDB 4oui) with *Bo*PDA-Co^{2+}. Residues from MT3-MT5 create the sides of the substrate binding pocket. The catalytic histidine (His 337) from MT5 is positioned close the substrate.

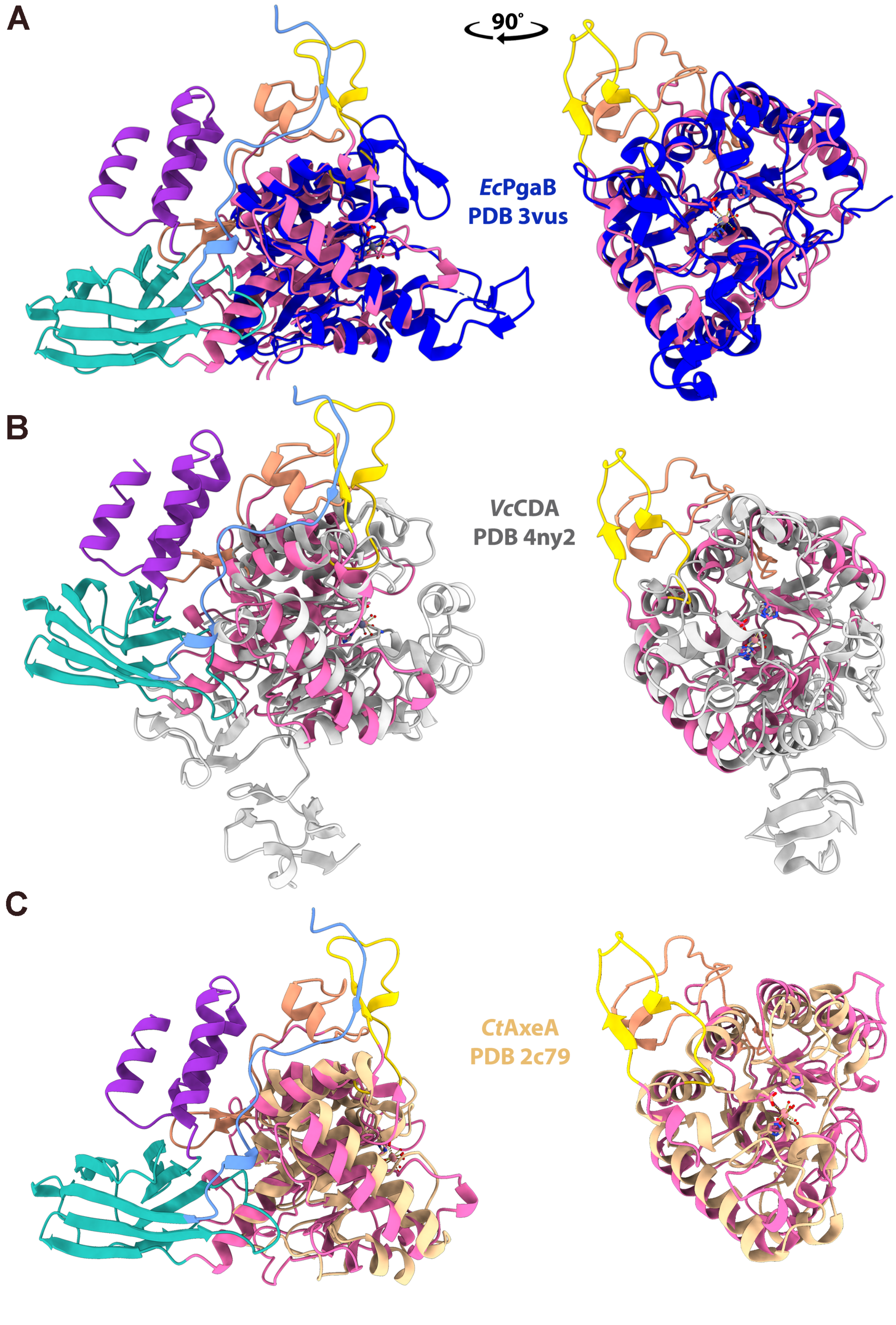
A
90°
EcPgaB
PDB 3vus
B
VcCDA
PDB 4ny2
C
CtAxeA
PDB 2c79

Figure S2. Overlays of *Bo*PDA with other CE4 Enzymes. Alignment *Bo*PDA with (A) *E. coli* poly-β-1,6-GlcNAc deacetylase (*Ec*PgaB; PDB 3vus; blue), (B) *Vibrio cholerae* chitin deacetylase (*Vc*CDA; PDB 4ny2; white) and (C) *Clostridium thermocellum* AxeA (*Ct*AxeA; PDB 2c79; tan). Superposition of *Bo*PDA with *Ec*PgaB and *Vc*CDA allowed mapping the location of MT3-MT5, and Loops 1-6 which surround the active site. *Bo*PDA is colored as in Fig 2. *Ct*AxeA is the only other example of a CE4 enzyme that uses a dyad (vs a triad) for coordinating the divalent cation, and its loops are relatively short compared to the other structures.

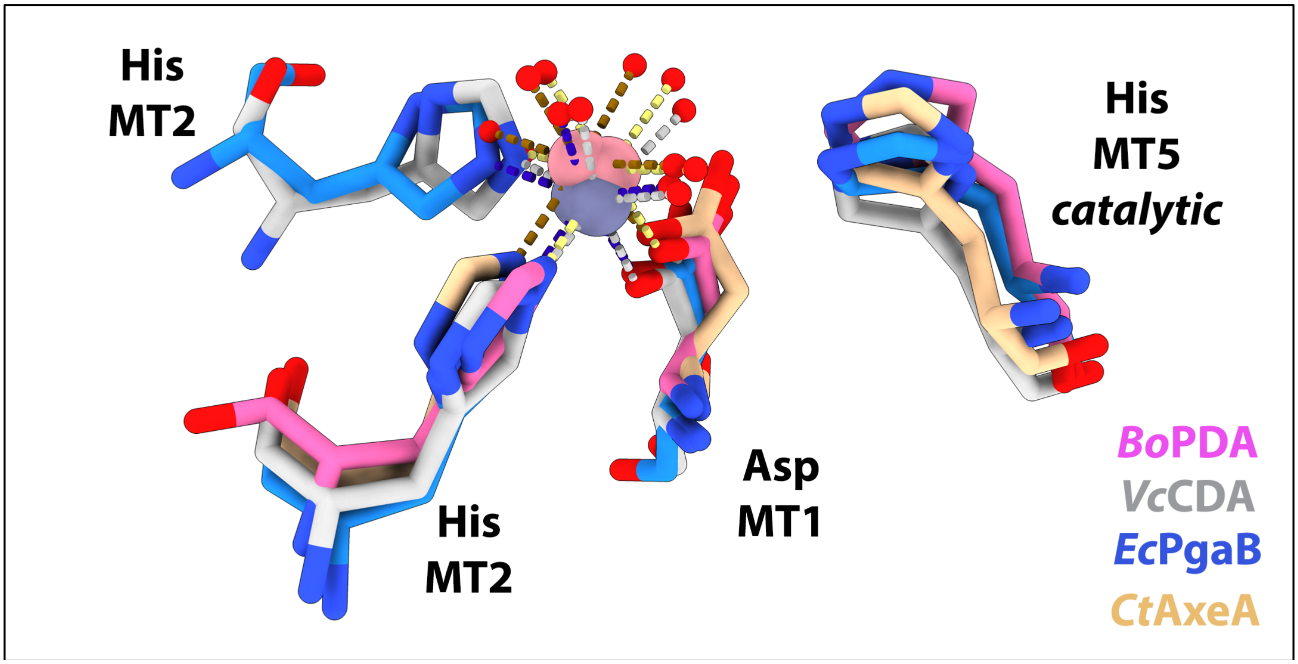

Figure S3. Active Site Overlays of CE4 Enzymes. The metal coordinating active site residues (MT1 and MT2) and catalytic histidine (MT5) active site residues were aligned from *Bo*PDA (pink), *E. coli* poly-β-1,6-GlcNAc deacetylase (*Ec*PgaB; PDB 3vus; blue), *Vibrio cholerae* chitin deacetylase (*Vc*CDA; PDB 4ny2; white) and *Clostridium thermocellum* AxeA (*Ct*AxeA; PDB 2c79; tan). Co2+ (pale pink; sphere) is bound to *Bo*PDA and *Ct*AxeA, while Zn2+ (gray; sphere) is bound to EcPgaB and *Vc*CDA. Bonds to the metal are shown in yellow (*Bo*PDA), blue (*EcPgaB*), brown (*Ct*AxeA) and white (*Vc*CDA). Waters are show as red spheres.

*Bo*PDA and *Ct*AxeA use a His-Asp dyad for coordinating the divalent cation, while all other known CE4 enzymes including *Ec*PgaB and *Vc*CDA use a His-His-Asp triad. *Bo*PDA and *Ct*AxeA only have one histidine in MT2, while *Ec*PgaB and *Vc*CDA have two hisitdines from MT2, as shown below. Additionally the position of the catalytic histidine from MT5 is conserved.

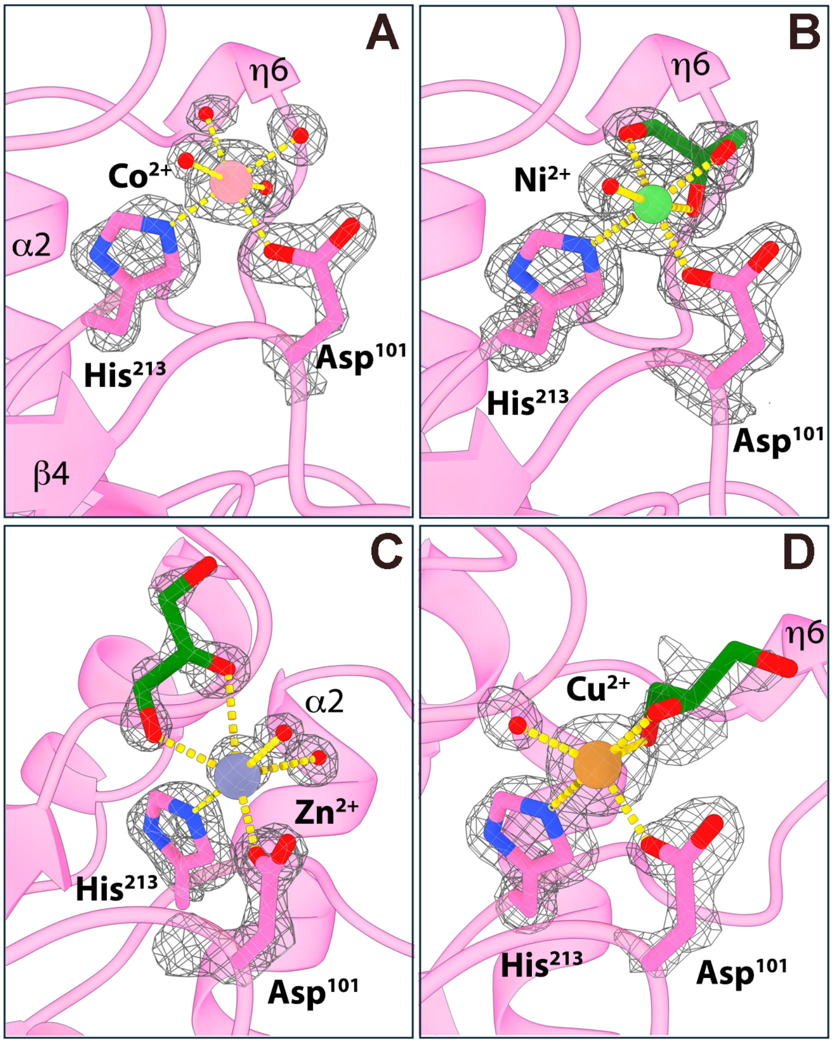

Figure S4. Active Site Composite Omit Maps. Composite omit map for each *Bo*PDA structure showing His[213] and Asp[101] dyad (pink, sticks, coordinating water molecules (red spheres), and glycerol molecules (green). Bound divalent cations are (A) Co^{2+} (pale pink; sphere), (B) Ni^{2+} (light green, sphere), (C) Zn^{2+} (gray; sphere) and (D) Cu^{2+} (brown, sphere) ions. Omit maps are contoured at 1.5σ in (B)-(D). (A) is identical to Fig 4E, with omit map contoured to 1.8σ. The glycerol molecule in *Bo*PDA-Cu^{2+} (D) is able to be modeled with two alternate conformations each at ~50% occupancy.

Figure S5. Carbohydrate Binding Assay SDS-PAGE. **(A)** Triplicate samples of each tested carbohydrate were prepared and run on 15% Tris-Glycine-SDS-PAGE as described in Materials and Methods. Coomassie brilliant blue stained gels were imaged by ChemiDoc (Bio-Rad) are shown below. Bands running near 50 kDa marker were quantified using ImageLab (Bio-Rad), as shown in the representative gel in (B). Those data are shown in Table S1 below.

A

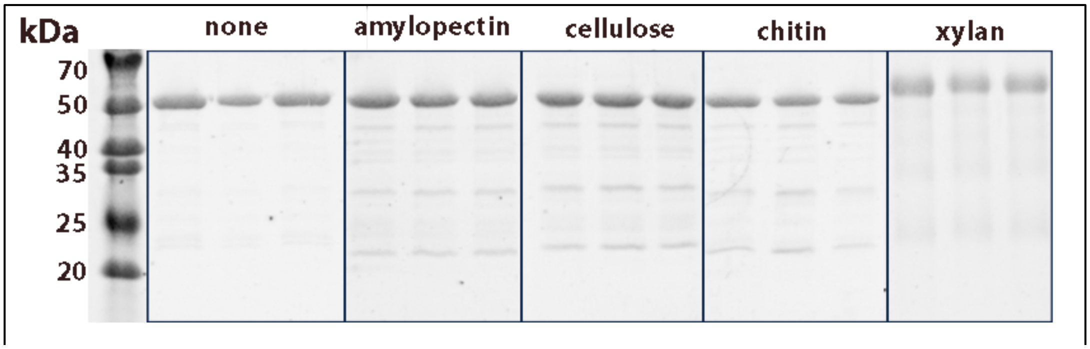

B

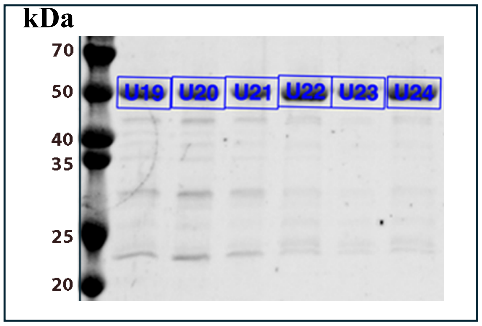

Table S1. Relative Binding Data for Carbohydrate Binding Assay SDS-PAGE.

	Replicates						
**Carbohydrate**	1	2	3	**Average**	**Stdev**	**Relative Binding**	**Error**
None	1.77E+06		1.64E+06	1.71E+06	9.76E+04	1	0.0572
Amylopectin	1.67E+06	1.43E+06	1.40E+06	1.50E+06	1.48E+05	0.879	0.0987
Cellulose	1.36E+06	1.52E+06	1.30E+06	1.39E+06	1.16E+05	0.817	0.0831
Chitin	1.45E+06	1.14E+06	1.00E+06	1.20E+06	2.30E+05	0.702	0.192
Xylan	1.12E+06	9.00E+05	9.93E+05	1.00E+06	1.12E+05	0.589	0.111

Note: Shaded columns are the values plotted in Fig 6A.

Table S2: *Bo*PDA deacetylase activity using Enzychrom™ Acetate Kit (Bioassay Systems)
No significant activity detected above control (no metal).

	Deacetylase Activity†	
**Metal**	***Chitin***	***Xylan***
No Metal	80.4 ± 6.55	126± 24.6
Co^{2+}	111.8 ± 16.2	103±9.79
Ni^{2+}	88.3 ± 12.7	87.2± 1.41
Zn^{2+}	101.3 ± 11.3	110± 12.1
† 20 mg substrate/acetic acid released (mM)		

Table S3. *Bo*PDA deacetylase activity with *p*-nitrophenyl acetate (*p*NPA)
No significant activity detected above controls (no protein or no metal) at concentrations tested.

	Absorbance min^{-1}				
**[pNPA] (mM)**	*No protein*	*No metal*	Co^{2+}	Ni^{2+}	Zn^{2+}
3.4	0.001±0.0014	-0.001±0.0014	-0.002±0.0000	-0.002±0.0000	-0.014±0.0085
13.6	-0.001±0.0042	-0.001±0.0014	-0.007±0.0042	0.0005±0.0007	0.001±0.0014
27.2	0.001±0.0014	-0.001±0.0014	-0.001±0.0014	-0.0035±0.0007	-0.01±0.0057
40.8	0.002±0.0000	0.004±0.0028	-0.003±0.0042	0.002±0.0000	0.001±0.0014
68	0.002±0.0028	0.004±0.000	-0.003±0.0042	0.0085±0.0007	0
170	0.013±0.0014	0.017±0.0014	0.013±0.0071	0.0145±0.0064	0.016±0.0000
340	0.053±0.0156	0.063±0.0099	0.0433±0.0076	0.021±0.0210	0.071±0.0042
